# Adrenal Lymphangioma Mimicking Renal Cyst: A Case Report and Review of the Literature

**DOI:** 10.1155/2013/136459

**Published:** 2013-11-03

**Authors:** Murat Akand, Mustafa Kucur, Pınar Karabagli, Ozcan Kilic, Bedreddin Seckin, Serdar Goktas

**Affiliations:** ^1^Department of Urology, School of Medicine, Selçuk University, Selçuklu, 42075 Konya, Turkey; ^2^Department of Pathology, School of Medicine, Selçuk University, Selçuklu, 42075 Konya, Turkey

## Abstract

We present a case of a 44-year-old female who was evaluated for left recurrent flank and abdominal pain. Abdominal ultrasonography demonstrated a large cystic mass on the upper pole of the left kidney. Magnetic resonance imaging showed a large, homogenous cystic mass measuring 8.5 × 9.5 cm with well-defined contours, being hypointense on T1-weighted images and hyperintense on T2-weighted images on the upper pole of the left kidney without a distinct plan between adrenal gland. According to clinical and radiological findings, surgical excision was carried out with a subcostal flank incision. Histologic and immunohistochemical examination demonstrates that the definite diagnosis is cystic lymphangioma of the left adrenal gland. Adrenal lymphangioma is a very rare lesion. This is the unique case of an adrenal lymphangioma considered as a renal cyst because of its radiological appearance.

## 1. Introduction

Adrenal cystic lesions are rare and mostly identified incidentally during radiological investigations or at surgery for unrelated reasons. Its incidence in autopsy series varies between 0,064 and 0,18%. Adrenal cysts are histologically classified into four main groups: endothelial cysts (45%), pseudocysts (39%), epithelial cysts (9%), and parasitic cysts (%7). Endothelial cysts are divided into two subgroups: lymphangiomatous and angiomatous cysts. Lymphangiomatous adrenal cysts are also known as adrenal lymphangioma (AL) [1]. Adrenal cysts are located on the suprarenal area and they can simulate renal, splenic, and pancreatic cysts. Despite the development in imaging techniques, the origin of the cyst could still not be distinguished in some cases [2].

We report a case of an AL, which is mimicking a renal cyst, with an emphasis on the symptoms, differential diagnosis, and treatment.

## 2. Case Presentation

**Figure 1:**
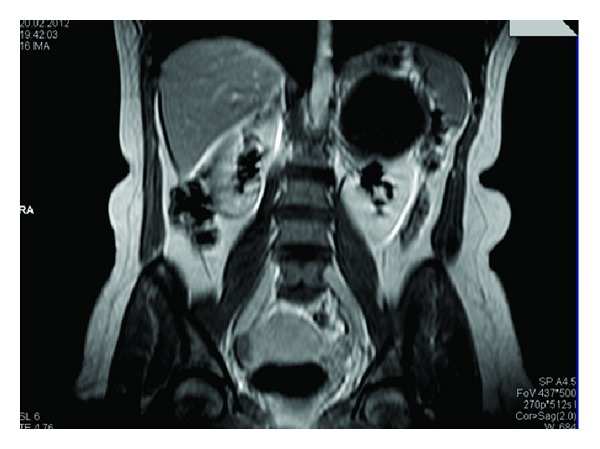
T1-weighted MRI image of adrenal lymphangioma in coronal section.

A 44-year-old female presented to the outpatient clinic with left recurrent flank and abdominal pain during the last 5 years. On physical examination, she was relaxed with palpation of the abdomen and was known to have a stabile hypertension and panic attack diseases. On laboratory evaluation, a normal urine analysis and normal hemoglobin, urea, and creatine levels were identified. Abdominal ultrasonography (US) demonstrated a large cystic mass on the upper pole of the left kidney. Magnetic resonance imaging (MRI) showed a large homogenous cystic mass measuring 8.5 × 9.5 cm with well-defined contours, being hypointense on T1-weighted images and hyperintense on T2-weighted images on the upper pole of the left kidney without a distinct plan between adrenal gland (Figure 1).

According to clinical and radiological findings, surgical excision was carried out with a subcostal flank incision. A cystic mass, approximately 10 cm in diameter, was identified on the suprarenal area without a distinct plan between left kidney and adrenal gland; therefore, the cyst was excised with a small part of the left adrenal gland without any intraoperative complications. Macroscopically there were osseous and cartilage-like tissues partly on the surface of the cyst wall. The cyst was filled with clear, nonviscous, brown colored fluid. After a 3-day hospital stay, the patient was discharged and the postoperative course was uneventful.

**Figure 2:**
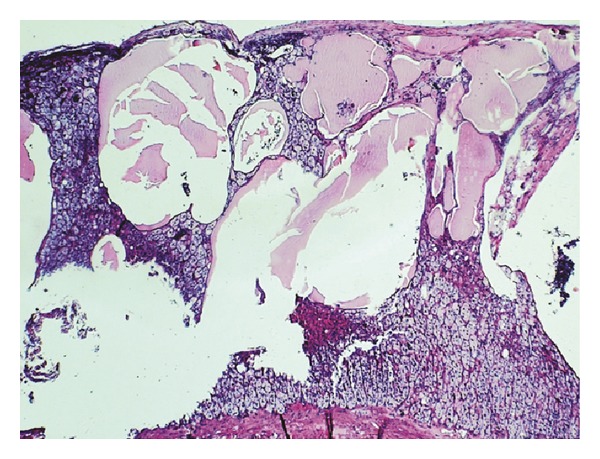
Multicystic architecture with dilated spaces lined by flattened lining cells (HE ×20).

**Figure 3:**
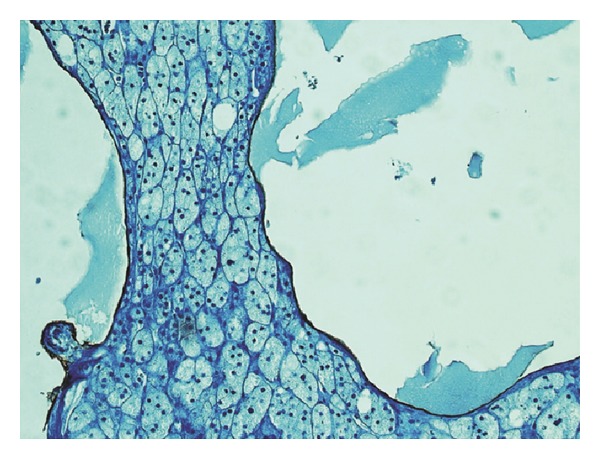
The flattened cells lining the cyst stained positive for D2-40 (×400).

Histologically, the cyst showed a typical multicystic architecture with dilated spaces lined by flattened, bland, simple lining. The cystic spaces occasionally contained proteinaceous material, lacking red blood cell content, and the cyst wall contained adrenal cortex (Figure 2). On immunohistochemical stains, D2-40 cytoplasmic staining was positive (Figure 3), whereas calretinin and CD34 were negative, thus, confirming their lymphatic nature. The definite diagnosis was cystic AL. Three months after the operation, the patient did not have abdominal pain. On abdominal US, left kidney was normal and the cyst was not existing on the suprarenal area.

## 3. Discussion

AL is a benign vascular lesion and a subtype of endothelial adrenal cysts. The first case of AL had been reported in 1965 and recently less than 50 immunohistochemically proven cases have been reported in the literature [3]. The largest series, reported by Ellis et al. in 2011, includes 9 immunohistochemically proven ALs [3]. 

The etiology and pathogenesis of AL are still unknown and matters of debate. The most favored theories are: malformation of lymphatic channels, ectasia of lymphatic channels, obstruction of proximal lymphatic channels, and a cystic degeneration in a hamartoma [4, 5].

ALs are macroscopically multilocular, thin-walled cystic lesions and are filled with nonviscous, clear, yellow-brown-colored fluid. Histologically, they are multicystic lesions formed by irregular dilated spaces lined with flattened, simple endothelial cells. Endothelial atypia has never seen in any of the lesions in the literature [1, 3]. Histologic characterization is not enough for definite diagnosis of AL, so immunohistochemical examination has to be made to prove the lymphatic origin of the cyst. D2-40 is a monoclonal antibody to the transmembrane mucoprotein which is expressed by lymphatic endothelial cells among others. D2-40 shows immunoreactivity to only lymphatic endothelium, so it is a specific marker for lymphatic origin [3].

ALs are usually asymptomatic. If they are symptomatic, symptoms are usually related to the mass effect and position of the cyst. The most common symptoms are abdominal or flank pain, gastrointestinal disturbance, palpable mass, headache, and arterial hypertension and palpitation especially in functional cysts [4–6].

The majority of the adrenal cysts are benign and non-functional. Neri and Nance reported 6 adrenal cyst cases of their own and reviewed over 600 cases, in which they detected that the malignancy incidence was 7% [7]. Functioning, and large cysts have a higher risk of malignancy. Because of the malignancy risk, definite diagnosis is very important for large and symptomatic adrenal cystic lesions [7].

On US, AL is a well-marginated, anechoic, cystic lesion typically located on the suprarenal area. There can be acoustic shadows and internal echoes, if calcifications or internal debris are present [1, 3, 8]. ALs are demonstrated as non-enhancing, low-density (0–20 HU) masses with smooth borders and thin wall (<3,5 mm) on computerized tomography (CT). On MRI, ALs are typically homogeneous, and hypointense on T1-weighted images and hyperintense on T2-weighted images with no internal enhancement. MRI is more sensitive for detecting intracystic hemorrhage and complicated cysts which is shown by high signal intensity on both T1 and T2 weighted images [9]. CT and MRI have high sensitivity in detecting adrenal cystic lesions. But large-sized cysts are usually confused with cysts from kidney, liver, spleen, and pancreas, thus causing a diagnostic dilemma [9].

In our case, MRI confirmed that the cystic mass were appeared that a part of the left kidney without a distinct plane between the kidney and the adrenal gland, so it was concluded that the the cyst originated from kidney rather than adrenal gland. We decided to resect the cyst surgically because of its large size and bothersome pain to the patient. To our knowledge, this is the unique case of an AL considered as a renal cyst because of its radiological appearance. But there are examples of misdiagnosis of other type of adrenal cysts in the literature [2, 5].

The subject of debate is which cysts need surgical intervention and which cysts need to be treated conservatively. The management of adrenal cysts and indications for surgery have to be based on oncological, endocrinological and clinical findings: (1) oncological indications: large cysts (5 cm or more in diameter), high density on unenhanced CT (>10 HU), delayed wash-out of the contrast medium, significant growth of the cyst on serial imaging, (2) Functional cysts, (3) Symptomatic cysts and cysts which can cause complications such as hemorrhage, rupture, or infection [10]. Small asymptomatic nonfunctioning cysts should be treated conservatively. If significant growth of the cyst or a functional status is detected during the follow-up, surgical excision is needed [10].
